# Community-based organizations’ experiences in using a digital resource referral platform (ConnectedNest) to support cancer patients

**DOI:** 10.1007/s00520-026-11425-z

**Published:** 2026-11-10

**Authors:** Eduardo R. Osegueda, Kris Newcomer, Allison L. Iwan, Alique Topalian, Helen M. Parsons, Abbie Begnaud, Pinar Karaca-Mandic, Dori Cross, David Haynes

**Affiliations:** 1 School of Public Health, Health Policy & Management, University of Minnesota, Minneapolis, USA; 2 School of Public Health, Epidemiology & Community Health, University of Minnesota, Minneapolis, USA; 3 XanthosHealth, Minneapolis, USA; 4 School of Public Health, University of Minnesota, Minneapolis, USA; 5 College of Medicine, University of Cincinnati, Cincinnati, USA; 6 Medical School, Department of Medicine, University of Minnesota, Minneapolis, USA; 7 Carlson School of Management, University of Minnesota, Minneapolis, USA; 8 Institute for Health Informatics, University of Minnesota, Minneapolis, USA

**Keywords:** Health-related social needs, Community resource referral platform, Cancer survivorship, RE-AIM framework

## Abstract

**Background:**

Over 8 million US patients with cancer experience health-related social needs (HSRNs) (e.g., food, transportation). Despite the clear importance of addressing HRSNs, there remains a gap in the IT-infrastructure allowing providers to monitor and facilitate patient connections to external resources. We created a community resource referral platform (ConnectedNest) to help connect cancer survivors with community-based organizations (CBOs). The goal of this study was to better understand the feasibility of implementing CRRPs into cancer-focused CBOs.

**Methods:**

After piloting ConnectedNest, we invited 13 CBOs to participate in semi-structured interviews to learn from their experience. Two qualitative researchers used a directed content analysis approach to identify insights and key recommendations for improving engagement based on constructs from the RE-AIM Framework.

**Results:**

We identified barriers and facilitators describing their (1) adoption of the ConnectedNest technology, (2) reach of clients, (3) implementation of ConnectedNest, and (4) long-term maintenance of the technology. CBOs varied in both in their organization’s programming and target populations. Participants reported alignment between ConnectedNest workflows and organizational roles as key factors for implementation success. All CBOs expressed interest in continuing to use ConnectedNest. Key recommendations include improving data integration and enhancing the caregiver-user experience.

**Discussion:**

CBO programming breadth and the size of their target client population largely determined their experience during the pilot study. Community resource referral platforms can support CBOs by clearly distinguishing unique programs and eligibility requirements within the platform.

**Conclusion:**

Community resource referral platforms have potential acceptability among cancer-focused CBOs. Co-creators see the potential value of these tools.

**Supplementary information:**

The online version contains supplementary material available at https://doi.org/10.1007/s00520-026-11425-z.

## Introduction

“Canceris a part of our life, but it’s not our whole life.”—Nick Procak, Melanoma Survivor [1]. Individualswith cancer often face numerous health-related social needs (HRSNs, e.g., transportation, food insecurity, financial burden, social isolation) that extend beyond the care traditionally provided in the medical setting [2–4]. Over 8 million cancer survivors in active treatment face potential interruptions to their treatments and clinical complications due to having unmet HRSNs that lower survival and quality of life and increased cancer disparities [2, 5–7]. However, the majority of oncologists state they have limited capacity to meaningfully engage their patients to address HRSNs due to barriers such as staff and provider time constraints and inaccessible support programs [4, 5].

There is an opportunity for developing and implementing community resource referral platforms (CRRPs) that connect patients, healthcare systems, and community-based organizations (CBOs) that provide services to address HRSNs [8, 9]. CRRPs are a potential solution to bolster the capacity of oncology care teams to more efficiently screen and assist patients with HRSNs [8]. However, there are significant barriers to implementing these systems in healthcare and community settings such as low CBO and patient engagement [10]. A recent study by Fichtenberg and colleagues that examined one such platform found that the majority of CBOs logged onto the platform less than once per month and that CBOs would value if the platform provided new ways for the CBO to learn about other resources in the community and facilitation of referrals [10].

To address CBO and patient engagement, we developed ConnectedNest, a patient and community-centered CRRP that connects individuals affected by cancer (people with cancer and their families) to cancer-focused CBOs [7, 11, 12]. ConnectedNest consists of three unique interfaces. The first is EmpowerNest, a mobile app that allows patients to screen themselves for HRSNs and connect with community programs that address those needs. The second is CommunityNest, a HIPAA-compliant web portal that allows CBOs to list their programs, communicate with patients, and make referrals to other CBOs and programs on the ConnectedNest network. At the time of the current study, the third interface that integrates into the electronic health system for clinical teams was under development.

ConnectedNest was developed using a human-centered design co-creation process and pilot tested with patients [7, 11]. During the 3-month pilot study of ConnectedNest with 41 individuals impacted by cancer and 13 CBOs, 39% of survivors were able to connect with resources provided by these organizations through the ConnectedNest app [13]. Building on the pilot study, qualitative study examines why cancer-focused CBOs adopt CRRPs and how engagement can be sustained. Using the Reach, Effectiveness, Adoption, Implementation, Maintenance (RE-AIM) framework [14], we analyzed changes in CBO engagement with CRRPs, offering insights into adoption and implementation. This study extends existing literature by incorporating perspectives from the early development of ConnectedNest and its creators.

## Materials and methods

### ConnectedNest feasibility study (previous)

We conducted a 3-month feasibility study of ConnectedNest from June to August 2023 with 13 CBOs recruited from the Minnesota Cancer Alliance (MCA). The MCA is a coalition of cancer-focused CBOs that serve people affected by cancer across the cancer continuum, funded by the Minnesota Department of Health through the Centers for Disease Control and Prevention.

We recruited 41 individuals affected by cancer through a continuous convenience sample. Participants included individuals on active treatment, those who completed treatment, and caregivers. ConnectedNest used a modified version of the Accountable Health Communities Survey [15] to screen participants for HRSNs and allowed them to search for programs that addressed their needs. Further details describing this mixed-methods study highlighting the feasibility and acceptability of ConnectedNest for addressing clients’ HRSNs can be found in Haynes et al. [7]

### ConnectedNest CBO implementation study (current)

#### CBOs setting and study design

The Implementation Study was conducted immediately following the ConnectedNest Feasibility Study to understand the compatibility and feasibility of integrating the ConnectedNest into CBO workflows. We followed the Consolidated Criteria for Reporting Qualitative Research (COREQ) Checklist (Appendix A) [16].

#### Recruitment and data collection

Through the MCA, we invited 13 organizations via email to participate in interviews; of those, 9 participated in 30-min semi-structured interviews. Author DC, who is a senior independent researcher, led three interviews; author ERO led the remaining six. Authors KN and DC attended eight and three interviews, respectively with two of these interviews overlapping (i.e., all three interviewing authors attending). Author ERO is a man while authors KN and DC are both women researchers. Author ERO is a PhD student with previous qualitative training and experience in conducting interviews and focus groups, author DC is an experienced qualitative researcher and an associate professor, and author KN is the community coordinator for XanthosHealth with previous experience as an executive director of a local cancer-focused CBO. Prior to the current study, authors ERO and KN had working relationships with all participants by nature of their roles at XanthosHealth. Author DC had no previous relationship with any of the participants. To establish rapport, all interviewers gave brief introductions and allowed participants to introduce themselves. Interviews were open only to participants and researchers (i.e., non-participants were not present). No repeat interviews were completed.

We developed our interview guide using the RE-AIM Framework as an organizing structure. We aligned CBOs experiences to RE-AIM constructs during the pilot study (Table 1) [17, 18]. The “Effectiveness” domain was not included in this analysis because the pilot study already evaluated it. The current qualitative study explores CBO experiences during the ConnectedNest pilot study. Reach was operationalized as CBO-reported client engagement levels to better align with the current implementation study. This definition is different from the traditional RE-AIM definition. Across domains, respondents were asked about facilitators and barriers shaping their pilot experience and recommendations for future refinement/scale. All participants were specifically asked to provide feedback on things that are going well with their use of ConnectedNest as well as areas where ConnectedNest could improve to elicit constructive feedback (Appendix B). Additionally, interviewers repeatedly encouraged participants to provide constructive feedback throughout the interview [19]. Interview guide questions were developed by authors EO and DC; this process was kept independent from other XanthosHealth authors.

**Table 1:** ConnectedNest community-based organizations from pilot study

CBO identifier	Population served		HRSN(s) served and example programs	Mission statement and description
CBOs participating in current qualitative study				
One	Any cancer	Cancer survivors	Physical activity Individual weight training Small group training	“Offers 8 free one-on-one sessions with a qualified personal trainer. The program is individualized for a person’s schedule and abilities. Our mission is to help people who have cancer, or who have had cancer, become as healthy as possible through strength training and other physical activity.”
Two	Any cancer	Cancer survivors and their families	Support Peer survivor support groups	“Provides free social and emotional support for anyone impacted by any type of cancer, including people living with cancer, and their families, friends, and caregivers. Our comprehensive program is built on five pillars of support: support groups, healthy lifestyle classes, educational opportunities, social connections, and resources & referrals—all designed to ensure that no one faces cancer alone. Through in-person and virtual offerings, we create a welcoming community where individuals can find connection, understanding, and hope.”
Three	Tumor specific	Cancer survivors and their families	Support Peer support group for spouses of ovarian cancer survivors Ovarian cancer survivor support gorups	“A statewide nonprofit organization serving those affected by ovarian cancer from throughout the Upper Midwest. [CBO 3] is dedicated to funding research, providing support to women and families, educating the medical community, and raising awareness.”
Four	Any-cancer	Cancer survivors and their families	Financial aid Emergency financial assistance	“Our mission is to offer relief to cancer patients and their loved ones through financial assistance, education, and emotional support.”
Five	Any cancer	Cancer survivors	Support Cancer doula care/survivor coaching	"We are a cancer thriver-led organization that offers compassionate, holistic support, including expert guidance and emotional support to individuals and families navigating the cancer journey. Always Navigating Your Cancer Journey TOGETHER.”
Six	Any cancer	Cancer survivors	Legal services Living will planning	“We engage the law to resolve complex challenges facing people and communities affected by cancer. Our mission and values exist in service to the vision we hold for a world that embraces equity and justice as fundamental to health.”
Seven	Tumor specific	Cancer survivors and their families	Support Lung cancer survivor support groups	“Founded in 2008 in the Twin Cities by a group of lung cancer patients who were concerned about the lack of awareness and research funding for lung cancer, the deadliest of all cancers. To raise awareness about lung cancer is to create a path toward improved wellness, earlier detection, and an increased survival rate. To this end, we fund lung cancer research, provide support for lung cancer patients, and educate the community.”
Eight	Tumor specific	Cancer survivors and their families	Support Colorectal cancer support groups	“Our core mission is to end colorectal cancer deaths by increasing screening and educating others about the signs and symptoms of this preventable disease.”
Nine	Any cancer	Caregivers and families	Support Caregiver podcast Caregiver peer support	“We are a nonprofit whose mission is to change the way caregivers of all kinds are seen and supported. They provide direct support to caregivers through podcasting, support groups, social media content, coaching, and caregiver gift boxes. In addition they provide education and tools to those in a position to support caregivers such as medical students and professionals, faith leaders, and community members.”
CBOs not participating in current qualitative study				
Ten	Any-cancer	Caregivers, families, and cancer survivors	Housing. Education Hope Lodge Cancer survivorship education	[Ten] provides a free home away from home for cancer patients and their caregivers. More than just a roof over their heads, it’s a nurturing community that helps patients access the care they need. Each [Ten] community offers a supportive, homelike environment where guests can share a meal, join in the evening’s activities, or unwind in their own private room. Patients staying at [Ten] must be in active cancer treatment
Eleven	Tumor-specific	Cancer survivors, caregivers, and their families	Support, Education Peer support for breast cancer Survivor mentorship	[Eleven] provides free, personalized one-to-one support for individuals who have been impacted by a breast cancer diagnosis. Our unique mentoring program extends the patients’ support network beyond their family and medical team—we connect them with a Peer Mentor Guide who shares a similar experience and lifestyle. Whether breast cancer has just been diagnosed, the person is in the midst of treatment, or treatment is fully completed, [Eleven] will offer guidance, support and hope
Twelve	Any-cancer	Caregivers, families, and cancer survivors	Support, Mental Health	[Twelve] is an unparalleled “family-focused” nonprofit dedicated to grieving Minnesota families since its inception in 2016. We are the only Family Grief Center in Minnesota providing no-cost care to all children, young adults, and adults who are grieving the death or terminal diagnosis of a family member. We are more than a counseling session; our model of care is truly exceptional, providing one-of-a-kind support logistically, psychosocially, and financially. We realize each family member grieves differently and, therefore, has different needs. Our goal is to help determine those needs and provide the services and resources to compassionately address them. We believe that HOPE begins HERE
Thirteen	Any-cancer	Cancer survivors in active treatment	Food	Since 1986, [Thirteen] has delivered well over 13 million medically tailored meals to clients with HIV/AIDS, cancer, amyotrophic lateral sclerosis, multiple sclerosis, end stage renal disease, congestive heart failure and/or chronic obstructive pulmonary disease and their families at no cost

CBO participants were aware of the study objectives. All interviews were conducted via Zoom. Zoom’s automatic transcription was used for all interviews. As a quality check, transcripts were obtained using Rev Ai software and compared to ensure accuracy.

#### IRB approvals

The study was determined not to be human subjects research by the University of Minnesota Institutional Review Board.

#### Qualitative data analysis

Two members of the research team (ERO and DC) analyzed interview transcripts using a directed content analysis approach [20]. Author ERO’s conflict of interest (based on being a XanthosHealth employee) was mitigated by having an independent author DC assist with the coding and analysis process. We coded content based initially on RE-AIM constructs, reviewing transcripts to add additional codes based on emergent themes as well as specific tagging of facilitators, barriers, and recommendations reported by participants, and then met to reconcile differences in coding lists and ensure the consistency of coding applications. Both qualitative analysts met multiple times to finalize the coding list and to develop guidelines to ensure the consistency of coding applications. Both analysts independently coded 2 transcripts and met to reconcile. Agreement on these two transcripts was over 95%. Additionally, the transcripts of interviews led by authors ERO and DC were compared to assess whether ERO’s company affiliation influenced participant responses. Interviewees were also asked to provide suggested changes to ConnectedNest; these recommendations were mapped to reflect the client user process of engaging with ConnectedNest and the core issue these changes intend to resolve. High-level themes identified from interview data were shared with participants at a community roundtable where participants were invited to provide feedback.

## Results

### Participating CBOs: scope of services and target population* (*Table 2*)*

**Table 2:** CBOs classified by population and programming

		Cancer type	
		Any cancer	Tumor specific
Services provided	Broad services	2	3
	Specialized services	4	0

CBOs participating in the implementation study (*n* = 9) listed programs covering 8 different needs categories. Table 1 describes the population each CBO serves, the HSRNs they address, and their organization’s mission and description. Two organizations were local chapters of larger national organizations; the remainder were formed in Minnesota.

### Classification of CBOs by services offered

Participating CBOs varied in terms of the breadth of the services they provided and the specificity of the populations affected by cancer that they served (Table 2). We categorized them by two criteria: (1) services provided and (2) cancer populations served. Some organizations provide multiple support programs (e.g., resource navigation, survivor groups), whereas other CBOs provide relatively specialized services (i.e., focusing on one specific HRSN, like financial assistance or physical activity). The second criterion identified CBO target populations. Some CBOs focused on providing services to individuals with specific tumor-sites (e.g., breast cancer), whereas others served anyone with cancer. Table 2 shows that CBOs who provided broad services tended to provide those services to specific cancer populations. Conversely, CBOs who provided specialized services did so for the entire cancer community. One pattern that emerged from the qualitative data was that CBOs providing rather niche services and serving survivors affected by any cancer reported experiencing higher levels of client engagement during the pilot phase.

### Barriers and facilitators to implementing ConnectedNest from the CBO perspective

Table 3 shows barriers and facilitators to implementing ConnectedNest from the CBO perspective.

**Table 3:** Barriers and facilitators to implementing ConnectedNest from the CBO perspective

RE-AIM domain	Definition	Emergent themes
Adoption	The motivating factors and reasons CBOs adopted the technology and participated in the ConnectedNest Feasibility Study	*Facilitators*: Finding partners, finding patients/survivors to serve *Barriers*: Not identified
Reach	The engagement of existing clients on ConnectedNest and the management of digital referrals	*Facilitators*: Ability to organize programs around distinct HRSN categories; CBOs leveraging their staff-client relationships to enroll survivors onto ConnectedNest *Barriers*: Uneven distribution of programs across HRSN categories; survivor capacity and readiness to connect to services; lack of catchment areas
Implementation	The intake and management of clients and use of intra-app functionalities	*Facilitators:* ConnectedNest serves as a central information repository; technology serves as a cue to action for survivors to find resources *Barriers:* Time crunch for CBOs to respond to client requests for services
Maintenance	The CBO’s long-term vision and perceived sustainability of the ConnectedNest technology within their organization’s operations	*Facilitators:* Unanimous CBO interest in continuing to use the platform. Comfort with key ConnectedNest functionalities *Barriers:* Changes are needed to encourage caregivers to find resources for themselves (e.g., caregiver support groups)

### CBO Motivation to participate in ConnectedNest (adoption)

CBOs consistently reported they adopted ConnectedNest for two reasons: (1) to find new clients and (2) to find additional organizational partners. Some CBOs viewed participating as an opportunity to increase awareness about their organization and services. Another benefit was improved community connection, as ConnectedNest provided up-to-date information about CBOs programs:

When I came to CBO 4… I knew some of the organizations and programs in my mind, and my predecessor had a resource list… but there’s always Googling, what’s new, what’s out there. Programs are always adding and dropping off, and we have clients who will be in locations that maybe we don’t have a lot of folks coming from… So yes, that’s one aspect of how I could see this would be helpful in that. - CBO 4.

### Digital referrals of clients to CBOs (reach)

Reach was assessed as the levels of client engagement reported by CBOs during interviews. Programs offering broader HRSN categories and tumor-specific resources reported experiencing higher levels of engagement. For these organizations, there was a natural synergy between the services provided and how their organization was presented in ConnectedNest.

[We offer] very specific things, and we were like the only program that, to my knowledge, was an adaptive fitness training [program]. So it’s like, automatically, they’re coming right to us. - CBO 1.

Reach was impacted by two factors: technological constraints and client readiness. During the study, the CBOs and programs made available to survivors were filtered based on a modifiable radius (e.g., 10 miles). CBOs noted the incompatibility of this feature for reaching the rural and out-of-state clients they serve who travel into the Twin Cities for treatment. Additionally, reach is impacted by client readiness to seek or receive services. Lack of readiness can be an especially formidable barrier early on in a survivor’s cancer diagnosis, as highlighted by the quote below describing the challenges of engaging survivors early in their journey:

Pre-COVID… you’re given all the resources with the diagnosis and a lot of time we’re in that [diagnosis/resources] folder, but we are overlooked… They’re [survivors] not going to reach out to us when they’re newly diagnosed most of the time. - CBO Two

### Client management and resource awareness (implementation)

One key strength CBOs saw in implementing the ConnectedNest platform into their organizational operations was providing a single platform for managing clients. CBO 3 suggested that ConnectedNest may help clients connect with the services they need by ensuring that HRSNs do not fade into the cracks amidst the chaos of treatment and life:

[Patients] get caught up with treatment and they forget about you because then it goes to the side because they’re just going through the motions. And so my thoughts was, maybe she saw my name like, ‘Oh gosh! I forgot I reached out to her,”... and then that [ConnectedNest] maybe triggered her to say, ‘Okay, I wanna reach back out to you to get connected.’ - CBO Four

Another strength of ConnectedNest was its ability to act as a resource repository to raise awareness for both CBOs and clients. This could help CBOs more easily share information internally about their clients’ needs and resources available to them in the community:

was meeting with] our board of directors, and they were like, how do you keep all this *information? Like, if something happened to you… where is it all going to be? [I think] it’s a great point that it is [all] on the app, right here.* - *CBO Three*

### Scaling up, future considerations, and recommendations (maintenance)

All nine interviewed CBOs expressed interest in continuing to use ConnectedNest for their organization’s purposes. Several participants expressed excitement about expanding the ConnectedNest community, both in terms of the number of CBOs participating on the platform and the number of individuals served.

I love ConnectedNest. I think you guys are doing a great job, and it will be great in five years. I really do appreciate that you do listen to all the community partners because I do think you need our buy-in, but it also really is for us which I really appreciate. I feel like you are creating it for us. - CBO Two

Additionally, one CBO shared that they were excited to continue using ConnectedNest because of the community created through our in-person/virtual hybrid town hall meetings.

Two of the other organizations I learned about through these meetings, and they came and participated in our patient summit because I learned about them in these meetings. they have such a unique and amazing service, you know, and we didn't know about them otherwise …it was a great way for us to connect and then connect our clients in a different way too than just through the app. - CBO Eight

There were several recommendations from CBOs to improve ConnectedNest so that it could better help CBOs reach survivors using the platform. These included suggested changes to the technology and insights into how to maintain the CBO buy-in for ConnectedNest as the platform grows. One technical suggestion was to find ways to increase the ease of exporting and importing client data to other data interfaces:

[We had] close to 450 clients this year [total for organization including outside referrals]… [engaging clients] through the app, it’d be a little time consuming because in the app [you] need to go one by one. But if there was a way to export a [mass] list or something, that would be helpful. - CBO Four

On the prospect of scaling ConnectedNest’s community, one respondent shared their perspective and preference to prioritize the inclusion of other cancer-focused CBOs when possible:

I think it’s fine to… especially in spaces where there aren’t cancer specific [resources]. If there were cancer-specific food shelves out there, I would advocate for those before others that were not [cancer-specific]. But there’s not, so I’d rather help people find the resource and keep them from having to jump from one tool to another to try and find these things. - CBO Seven

Similarly, another CBO emphasized the importance of establishing a feedback loop between ConnectedNest and its community. This would establish trust and engagement amidst technological and operational changes:

…having some kind of like a peer review process for adding new organizations. You want to have us in conversations to have our buy in, right? Otherwise, we won’t use it. - CBO Two

## Discussion

Navigating a cancer diagnosis and survivorship is a complex journey for survivors and their loved ones. Unmet HRSNs further complicate this care path and contribute to poorer outcomes among cancer patients and contribute to cancer disparities [2, 21]. Still, more needs to be done to meaningfully integrate HRSNs into oncology care. Thus, we developed and piloted ConnectedNest, a CRRP, to facilitate connections between individuals affected by cancer and community-based organizations that serve them. Recent literature highlights, however, that CRRPs often struggle to maintain active CBO engagement. Thus, the current qualitative study applied RE-AIM to understand facilitators and barriers to CBO engagement with ConnectedNest.

### The importance of value alignment

Initial adoption of ConnectedNest and CBO’s intention to maintain their use of the technology long-term was driven by their desire to use ConnectedNest to find and serve new clients and form new interorganizational partnerships. This value alignment of CBO needs and ConnectedNest’s mission coupled with voluntary adoption of the technology likely played a role in CBO engagement. Without this value alignment, CRRPs struggle to maintain engagement. Fichtenberg and colleagues found that the CBOs with the lowest levels of engagement with the technology were those that did not view the platform as an efficient way to reach patients [10]. Additionally, they found that CBOs viewed the COVID-19 pandemic as a barrier limiting their engagement with the CRRP [10]. In contrast, COVID-19 and its impact on in-person client recruitment and closer interorganizational partnerships was viewed as a catalyst for change by the CBOs voluntarily adopting ConnectedNest. Additionally, Fichtenberg et al. found that CBOs reporting discomfort and uncertainty using CRRP functions such as digital referrals were less engaged with the technology [10], and less likely to continue using the CRRP. Our findings echo these results as CBO participants shared their intention to continue using ConnectedNest and their comfort of using ConnectedNest’s e-referrals. Ultimately, CRRP tech literacy among CBOs reinforces adoption and facilitates the potential for long-term maintenance and integration of CRRPs.

### The importance of hyperlocal CBO network

Adopting CBOs also intended to use ConnectedNest to find new partners. One interviewee shared that participating in the study led their CBO to form a new partnership. This newly formed connection was between a national-level tumor-specific support organization and a smaller, any-cancer specialized service provider. This example serves as another opportunity for CRRPs: the ability for large national-level cancer support organizations to be connected with smaller, more locally integrated service providers.

As ConnectedNest scales up, interorganizational partnerships and personalized client experience provide CBOs with opportunities for warm handoffs. Warm handoffs done through ConnectedNest’s digital features may facilitate easier client-organization connections, thereby reducing the time and emotional burden clients face when trying to access resources [22] and effectively addressing HRSNs [23, 24]. This is a direct improvement upon many other health system initiatives to address patient HRSNs; one recent review of 41 studies found that only two interventions incorporated warm handoffs between healthcare providers and CBOs [25].

### The importance of whole patient support

ConnectedNest must balance increasing the breadth and distinction among services offered to meet the vast needs of survivors as technology expands. The National Cancer Center Network’s Distress Thermometer comprises over 40 unique HRSN categories [26]. Cancer survivors’ needs encompass everything from managing their daily lives to maintaining their dignity and individuality throughout their cancer journey [27]. Careful design of CRRPs can help simplify and humanize the resource search process. Incorporating Human-Centered Design principles such as a participatory and iterative design process can ensure cancer survivors that CRRPs are designed to consider the entirety of their survivorship journey [28].

This study highlights the importance of ensuring that CBOs programs are found in categories best describing their services. CBOs with unique services listed on ConnectedNest in HRSN categories such as legal assistance and physical activity saw the highest levels of engagement. Conversely, CBOs offering programs in relatively saturated HRSN categories (e.g., support) and to people impacted by tumor-specific cancers (e.g., breast) experienced lower levels of client activity. A challenge for CRRPs is thus to equitably integrate both unique service providers and more broad cancer support communities within a single interface. One solution is to incorporate additional nuance for broad service categories (e.g., distinguishing support groups from one-on-one survivor coaching). It is important to balance specificity with simplicity to not overwhelm survivors. Other CRRPs have as many as 27 unique HRSN areas [29]. Further research is needed to explore survivor and caregiver preferences regarding CRRP’s balance of specificity and simplicity. Additional research is also needed to further explore the pattern of program specificity and CBO-reported engagement as CRRPs like ConnectedNest expand to include more CBOs, and particularly CBOs that provide specialized services for tumor-specific populations (a group not observed in the current study).

CRRP developers may also improve the patient and CBO experience by providing CBOs technical assistance training. As noted by some interviewees, informal community roundtable discussions about technology updates and open conversations can lead to new interorganizational partnerships. These partnerships equip large support organizations with specialized service providers to refer their clients to, bolstering their role as referral powerhouses. Further research is needed to better understand how CBO characteristics impact how they use CRRPs.

### Limitations

This study has several limitations. First, given the relationship between participants and authors EO and KN, we were concerned about positive response bias; however, we did receive critical feedback. Additionally, the CBOs who volunteered to participate in interviews are a self-selecting group and may have a more positive outlook on ConnectedNest than those CBOs that did not participate in the study. Secondly, this study describes and analyzes CBO staff member experiences from a pilot study. Our sample size of 41 people affected by cancer resulted in limited client interactions, particularly for organizations with narrower target populations, as several CBOs did not form any new connections through ConnectedNest during the study. Thirdly, this platform is designed for those impacted by cancer. Thus, the perspectives of ConnectedNest’s CBOs might not directly apply to CRRPs serving other patient populations.

## Conclusion

CBOs play a critical role in whole-person cancer care by helping survivors meet their basic needs and maintain their sense of identity and peace through their cancer journey [30]. Implementing CRRPs could improve the process of resource connection for health systems, patients, and CBOs. In this study, ConnectedNest functionality engaged CBOs as both technology recipients and resource deliverers [31]. Like all CRRPs, ConnectedNest’s ability to support cancer survivors and their families relies heavily on the engagement and buy-in of CBOs who actively list programs and complete referrals on that platform [8, 10]. Our study found that while cancer-focused CBOs voluntarily adopted ConnectedNest for similar reasons the ways in which they experience and implement the technology varied. CRRP developers should be responsive to the needs and experiences of their CBO partners.

## Supplementary information

Below is the link to the electronic supplementary material.

ESM 1 (PDF 101 KB)

ESM 2 (PDF 485 KB)

## Data Availability

No datasets were generated or analyzed during the current study.
