# Supplementary material for: Community-based organizations’ experiences in using a digital resource referral platform (ConnectedNest) to support cancer patients
Source: Support Care Cancer. 2026 Nov 10;34(12):1217. doi: 10.1007/s00520-026-11425-z (PMC13751745; doi:10.1007/s00520-026-11425-z)
Supplement: Supplementary file 1 [file 520_2026_11425_MOESM1_ESM.pdf]

The moderator will review the following at the beginning of CBO interview:

1) Introduce self and other study staff, if applicable
2) Thank participant
3) Provide the purpose of the interview
4) Review confidentiality and privacy

1) Introductions

   Hello, my name is [______], and I am from the University of Minnesota. Thank you for taking the time to be here today. The long-term mission of the ConnectedNest Platform is to improve cancer care by creating connections between people with cancer, their caregivers, and community-based organizations; facilitating these connections will help patients and their families get the help they need with the nonmedical challenges associated with a cancer diagnosis.

2) Thank participant

   Thank you for your organization's participation in the ConnectedNest Pilot. With your help we were able to create a community of 13+ organizations providing services for survivors and caregivers. We also thank you for taking the time to share your thoughts on the current technology. We want to make sure this platform is useful; please share your honest thoughts and opinions on the technology. This is a safe space. Anything said here will be kept confidential.

3) Provide purpose of the interview

   This interview should take no more than 30 minutes of your time. We will ask about your experience connecting with and serving members of your community via the ConnectedNest platform. We want to know what things are going well as well as areas for improvement and any suggestions you may have so that we can better support your use of the platform.

4) Review confidentiality and privacy

   We are recording this discussion, so that we don't miss anything. The only people who will ever hear this recording are study staff. The recording will be deleted when the study is over.

   Your participation is completely voluntary. You may withdraw from this interview at any time for any reason.

   Do you have any questions before we begin?

Interview Guide
(CBO Participation Reasoning/Facilitators/ADOPTION)

1. **What drew your organization to participate in the ConnectedNest Pilot Study?**

(Recruitment/REACH) -> Note: not all CBOs are involved in recruitment.

2. **Describe your organization's experience with recruiting your community members into the ConnectedNest study.**
    a. Describe any particular challenges in recruiting study participants.
    b. Describe anything that helped with recruitment.
    c. Did any of your community members comment or have specific questions about registering within ConnectedNest?

(Intake & Management/IMPLEMENTATION)

3. **How was your experience in connecting with your existing clients using the ConnectedNest platform? Did you find any new clients outside of your existing survivor network?**
    a. If no: What challenges did you face in expanding your survivor network with the technology?
    b. If yes: What features or processes worked well in supporting these connections? What worked not so well?

4. **Describe your internal process in terms of managing intake and connections within the ConnectedNest platform? What resources did it take to manage your organization's presence on the app?**
    a. Did you ever change the number of programs listed by your organization on ConnectedNest? If so, did you add or remove programs; why?
    b. Did you ever change the capacity of your listed programs on ConnectedNest?
    c. Describe your organization's process closing the loop. How did you ensure that clients who connected with you virtually via ConnectedNest received the services they needed?
        i. Were there any specific challenges you came across?

5. **Did you refer anyone to another organization on the platform? Why or why not? Did you receive any referrals from other CBOs?**

  a. How did you utilize the Chat function on the platform?
  b. Are there any additional functions you would like to see added to the platform?
  c. How did you use the drop-down options for client demographics to better reach people fit for your programs?
      i. Are there any filters/criteria you would like added to determine program eligibility?
  d. How did you use the bookmark functionality to track client's connection and service receipt statuses?

(Sustainability and Moving Forward/MAINTENANCE)

6. **Would you want your organization to continue using the ConnectedNest interface after the study period? Why or why not?**
    a. If yes: Would you be willing to include non-cancer focused support organizations in the ConnectedNest community (e.g., a food shelf serving anyone in need of food).
    b. If not: what would be a deciding factor that would make ConnectedNest worth using?

7. **How well does the ConnectedNest platform align with your organization's mission? How well does it align with your organization's resources?***
    a. If not, what changes could be made to the platform to make things easier for organizations with fewer resources?
    b. What goals did you have coming into the ConnectedNest study?
        i. What factors helped you achieve your goals?
        ii. What challenges did you face using ConnectedNest?
